# Early lactate and glucose kinetics following return to spontaneous circulation after out-of-hospital cardiac arrest

**DOI:** 10.1186/s13104-021-05604-w

**Published:** 2021-05-13

**Authors:** Pedro Freire Jorge, Rohan Boer, Rene A. Posma, Katharina C. Harms, Bart Hiemstra, Bas W. J. Bens, Maarten W. Nijsten

**Affiliations:** 1 Department of Critical Care, University Medical Center Groningen, University of Groningen, PO Box 30.001, HPC TA29, 9700 RB Groningen, The Netherlands; 2 Department of Anesthesiology, Amsterdam University Medical Center, University of Amsterdam, Amsterdam, the Netherlands; 3 Department of Emergency Medicine, University Medical Center Groningen, University of Groningen, Groningen, The Netherlands; 4 Department of Anesthesiology, University Medical Center Groningen, University of Groningen, Groningen, The Netherlands

**Keywords:** Glucose, Lactate, Kinetics, Out-of-hospital cardiac arrest, Recovery, Return of spontaneous circulation, Cori cycle

## Abstract

**Objective:**

Lactate has been shown to be preferentially metabolized in comparison to glucose after physiological stress, such as strenuous exercise. Derangements of lactate and glucose are common after out-of-hospital cardiac arrest (OHCA). Therefore, we hypothesized that lactate decreases faster than glucose after return-to-spontaneous-circulation (ROSC) after OHCA.

**Results:**

We included 155 OHCA patients in our analysis. Within the first 8 h of presentation to the emergency department, 843 lactates and 1019 glucoses were available, respectively. Lactate decreased to 50% of its initial value within 1.5 h (95% CI [0.2–3.6 h]), while glucose halved within 5.6 h (95% CI [5.4–5.7 h]). Also, in the first 8 h after presentation lactate decreases more than glucose in relation to their initial values (lactate 72.6% vs glucose 52.1%). In patients with marked hyperlactatemia after OHCA, lactate decreased expediently while glucose recovered more slowly, whereas arterial pH recovered at a similar rapid rate as lactate. Hospital non-survivors (N = 82) had a slower recovery of lactate (P = 0.002) than survivors (N = 82). The preferential clearance of lactate underscores its role as a prime energy substrate, when available, during recovery from extreme stress.

**Supplementary Information:**

The online version contains supplementary material available at 10.1186/s13104-021-05604-w.

## Introduction

Under physiological stress, such as a bout of intense exercise or critical illness, the body generates a metabolic response characterized by hyperlactatemia and hyperglycemia [1, 2]. In the post-exercise period, healthy individuals are able to clear large amounts of lactate [3–5]. Exercise studies demonstrated that—when available—lactate is preferentially consumed in comparison to substrates such as glucose [3, 6, 7].

In critically ill patients, hyperlactatemia is a common occurrence, caused by a variety of factors essentially involving the increased production or diminished consumption of lactate [8]. During the recovery phase from critical illness, the restoration of normal lactate levels and the rate at which this occurs is associated with outcome [9–12]. Likewise, glucose levels and their recovery have been associated with outcome following out of hospital cardiac arrest (OHCA). Significant hyperglycemia and increased time to normalize glucose levels have been associated with poor outcome [13].

The period following return of spontaneous circulation (ROSC) after OHCA often reflects rapid restoration of normal circulation. Hyperlactatemia recovery after OHCA has been studied over time scales from 12 to 24 h, [10] but these kinetics have not been described for the first hours after OHCA.

We aimed to identify the kinetics of lactate and glucose in the first hours following ROSC after OHCA in patients who present with marked hyperlactatemia. We hypothesize that both glucose and lactate levels decrease fast after ROSC, but that the decrease in lactate occurs faster than glucose.

## Main text

### Methods

We performed a retrospective study of the patients presenting after OHCA to the emergency department (ED) of our principal tertiary referral hospital for the north eastern provinces of The Netherlands in a region with 750,000 inhabitants between 2006 and 2016. Patients being 18 years or older who achieved ROSC after cardiopulmonary resuscitation, and who were subsequently admitted to the ICU were eligible for our study. We included patients who survived the first 8 h of ICU admission, had an initial lactate level of ≥ 8 mmol/L, and had at least two lactate measurements within the first 3 h after ICU admission. We excluded patients who died before ICU admission, and for whom the cause of OHCA was not primarily cardiac. Only the last OHCA event of patients who suffered multiple OHCAs between 2006 and 2016 was analyzed.

We collected descriptive data such as age, sex, presence of diabetes mellitus, initial rhythm after OHCA, and whether pre-hospital BLS was performed from the ambulance registry and hospital information system. We collected all lactates, glucoses and arterial blood gas results measured between the first measurement at the ED and 8 h after presentation. Additionally, we compared data between hospital survivors and hospital non-survivors. This study concerning anonymized data was performed in accordance with the guidelines outlined in Dutch legislation, and the study was approved by the medical ethics committee of our institution (Medisch Ethische Commissie, UMC Groningen, METc 2015/488). Because this was a retrospective study of routinely collected data, informed consent was not required by our ethics committee.

Categorical data are presented as proportions. Continuous data are presented as means and 95% confidence interval (95% CI). Patient characteristics of hospital survivors and non-survivors were compared using the Student’s t-test for continuous data and the chi-square test for categorical data. No lactate or glucose values were interpolated or imputated. Changes over time for lactate and glucose were fit by mixed-effects models using the *lme4* package with a random slope and intercept on an individual level. An unstructured covariance structure was assumed. Type of laboratory measurement (lactate or glucose), time, and the interaction of time with the type of laboratory measurement were entered into the model as covariate. Both as fixed and as random effect, time was modeled as natural cubic spline with knots placed at 0.5, 2, and 5 h after ED presentation using the *splines* package. The knot locations were chosen to increase generalizability and were based on tertiles. In two separate models, lactate or glucose patterns over time were compared for hospital survivors and non-survivors, and thus the interaction of hospital survivorship with time was entered as covariate. Bootstrap confidence intervals for the time to reach 50% of the initial value for lactate and glucose were generated via 1000 bootstrap samples that were obtained from the mixed-effect model using the *bootmer* function. Absolute decrease (in mmol/L/h) in lactate and glucose levels were determined by creating a contrast matrix within the *Epi* package. Data were analysed using R version 3.6.2 (R Foundation for Statistical Computing, Vienna, Austria).

## Results

We included 155 patients in the analysis. Eighty-two percent were male and the mean age was 59 ± 14 years. The majority (81%) of patients had ventricular fibrillation as initial rhythm and 12% of patients had documented diabetes in their previous medical history (See Additional file 1: Table S1).

**Fig. 1:**
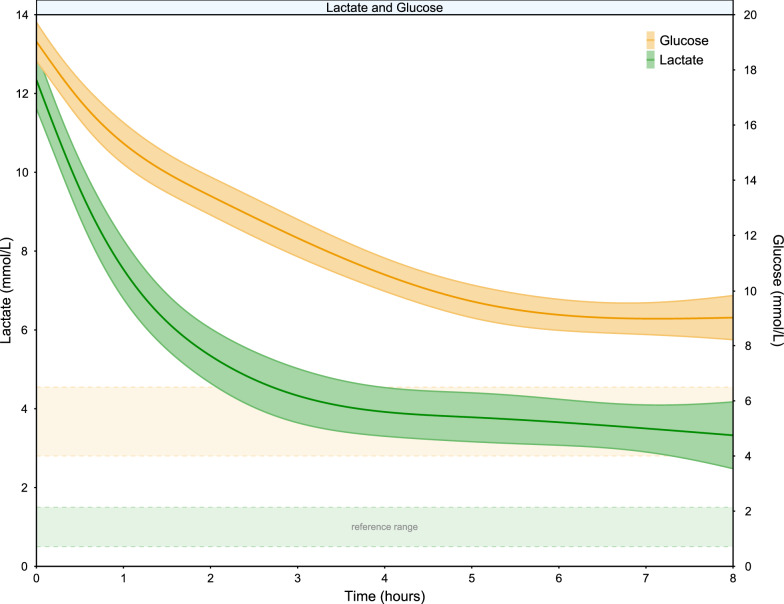
Evolution of lactate and glucose levels for 155 patients in the first 8 h after OHCA. It can be observed that lactate shows a more acute initial decrease compared to glucose

Mean (95%CI) initial lactate was 12 mmol/L (11.6–12.7 mmol/L). The lactate concentration decreased 9.0 mmol/L (95% CI [8.0–10.0 mmol/L]) in the first 8 h after presentation and the estimated time to reach 50% of initial lactate was 1.5 h (95% CI [0.2–3.6 h]) (Table 1). The lactate kinetics over the first 8 h after ROSC are depicted in Fig. 1.

**Table 1:** Lactate and glucose variables after OHCA

	Lactate		Glucose		Between-group difference		P-value
	Mean	95% CI	Mean	95% CI	Mean	95% CI	
Initial value (mmol/L)	12.2	11.6–12.7	18.8	18.0–19.6	–	–	
Measurements per patient within 8 h	5.4	5.1–5.8	6.6	6.3–6.9	1.14	0.7–1.6	< 0.001
Absolute decrease over 8 h (mmol/L)	9.0	8.0–10.0	10.0	9.1–10.9	–	–	–
Relative decrease over 8 h (%)	72.6	68.0–77.2	52.1	48.8–56.4	20.5	15.7–25.3	< 0.001
Time to reach 50% of the initial value (hours)^a^	1.5	0.2–3.6	5.6	5.4–5.7	4.5	2.8–6.0	< 0.001

Lactate and glucose parameters for the whole sample. Lactate decreases faster compared to glucose in the early phase after OHCA. ^a^ Basic 95% confidence interval obtained after bootstrapping the mixed-effect model for a thousand times

Mean (95% CI) initial glucose was 18.8 (18.0–19.6) mmol/L. The glucose concentration decreased 10.0 mmol/L (95% CI [9.1–10.9 mmol/L]) in the first 8 h after presentation and the estimated time to reach 50% of initial glucose was 5.6 h (95% CI [5.4–5.7 h]) (Table 1). The glucose kinetics over the first 8 h after ROSC are depicted in Fig. 1.

Lactate displayed a more acute decrease in the very early phase compared to glucose. Glucose showed a more linear and protracted recovery (Fig. 1). The time to reach 50% of the initial value was faster for lactate compared to glucose (lactate 1.5 h vs glucose 5.6 h) (Table 1). Also, in the first 8 h after presentation lactate decreases more than glucose in relation to their initial values (lactate 72.6% vs glucose 52.1%) (Table 1).

Hospital survivors had significantly faster decreases in lactate compared to hospital non-survivors (Additional file 1: Table S2). There were no differences in glucose kinetics between hospital survivors and hospital non-survivors (Additional file 1: Table S3).

**Fig. 2:**
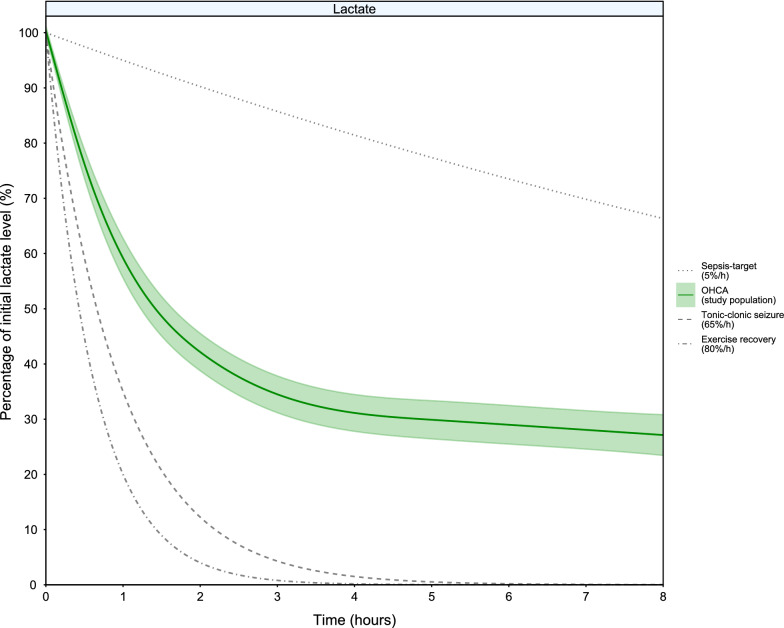
Evolution of fraction of initial lactate in different settings in the first 8 h after event. Here we compare the changes in lactate levels in our patients after OHCA with the lactate levels after exercise [5], after a tonic–clonic seizure [14], and with the desired lactate decrease in the setting of sepsis of 5% per hour [12]. Shaded area indicates 95% confidence interval

Early relative lactate decrease is faster compared to a rate of 5% for sepsis [21]. It is however lower than the rate observed in athletes after heavy exertion [5] and after the cessation of tonic–clonic convulsions [14] (Fig. 2).

The recoveries of other metabolic parameters (pH, pCO2, base excess) are shown in the Additional file 1: Figures S2–S4. We also depicted individual curves of the kinetics of lactate, glucose, pH, pCO_2_ and base excess (Additional file 1: Figures S5–S9).

## Discussion

In patients admitted to the ICU after OHCA, lactate decreased considerably faster than glucose. While lactate reached 50% of its initial level within 1.5 h and had a 73% decline in the first 8 h, glucose reached 50% of its initial level within 5.6 h and declined by 52% in the first 8 h. Although it was not our primary objective, we also observed that hospital survivors had a higher relative decrease of lactate compared to hospital non-survivors, while we did not find such association for glucose during the first 8 h (Additional file 1: Table S2 and S3—between group differences). Early lactate decrease after ROSC after OHCA was fast and approached lactate decreases reported during recovery after exercise and tonic–clonic seizure (Fig. 2) and much faster than a recovery of 5 to 10%/h that is considered desirable during sepsis treatment. Our hospital blood gas analysis routine provided many glucose and lactate measurements and thus this study was enriched by a relatively large number of measurements in a short period of time. To our knowledge this is the first study to describe the very early kinetics of lactate and glucose after OHCA on such high resolution.

After physiological stress such as seizures or strenuous exercise, lactate levels can be higher than 15 mmol/L [5, 14] due to generalized muscular contractions and strong adrenergic stimulation. Upon OHCA, the body enters a no-flow or low-flow state with accompanying hyperlactatemia and hyperglycemia as a result of glycolytic metabolism and strong glycogenolysis, which in turn result from hypoxia and strong endogenous and (subsequent) exogenous catecholamine and cortisol surges [1, 2, 15]. Rapid restoration of sufficient circulation and oxygenation enables many tissues to clear lactate by direct oxidative metabolism or conversion back to glucose through the Cori cycle [16]. Post-exercise or after seizures lactate can decrease by 40 to 90%/h [5, 14]. Such observations underscore the immediate metabolic adaptation by which lactate can be cleared in persons with intact or restored hemodynamics [5, 14]. The total lactate load in our patients just after ROSC was considerable and ranged approximately between 200 to 500 mmol assuming a distribution volume of 0.2 to 0.5 L/kg [17]. As indicated in Fig. 3, the early course of lactate decrease post-OHCA approached that of post exertion and post-seizure recovery [5, 14].

OHCA is also typically followed by marked hyperglycemia due to glycogenolysis and possibly later gluconeogenesis caused by the same adrenergic and adrenocortical responses that induce hyperlactatemia [18]. The slower correction of this hyperglycemia has also been shown to be associated with worse outcomes [12, 19]. Also, there is clinical evidence that administration of glucose solutions in the peri-arrest period is related to worse neurologic outcomes [20]. Whether intensive glucose regulation improves outcomes is still a matter of debate, due to higher risk of hypoglycemia which can also be harmful [21]. We recently published a large retrospective study showing that early normoglycemia combined with hyperlactatemia in critically ill patients is associated with increased mortality. We hypothesized that this is due to early dysfunction of hepatic and renal gluconeogenesis [22]. In patients with sudden cardiac arrest, the event is most likely not precluded by organ failure which could impair metabolism. Moreover, we assume that before most OHCA events, sufficient hepatic glycogen reserves are present, compatible with the markedly elevated circulating glucose levels that are rapidly generated.

The initial cellular energy deficit after OHCA is apparently not primarily corrected by glucose uptake but by uptake of lactate. The monocarboxylate transporter (MCT) family facilitates the bidirectional transmembrane transport of the lactate anion together with a proton [23]. Although the terms lactate and lactic acid are often used interchangeably, it is important to underscore that the MCT transports lactic acid, although lactic acid is essentially fully dissociated at (patho-)physiological pH’s (in Lactate^–^ and H^+^). The simplified stoichiometry of the two main fates of lactic acid is respectively

$$ {\mathbf{2\, Lactate}}^{{\mathbf{ - }}} {\mathbf{ +  2\, H}}^{{\mathbf{ + }}} {\mathbf{ +  6 \,O}}_{{\mathbf{2}}}  \to \left( {{\mathbf{full oxidation}}} \right){\mathbf{ \to 6\, CO}}_{{\mathbf{2}}}$$

and

$$  {\mathbf{2\,Lactate}}^{{\mathbf{ - }}} {\mathbf{ +2\, H}}^{{\mathbf{ + }}} {\mathbf{ \to }}\left( {{\mathbf{gluconeogenesis}}} \right){\mathbf{ \to 1\, Glucose}} $$

The cellular uptake of lactate as lactic acid also explains that the restoration of pH (Additional file 1: Figure S2) parallels changes in lactate in the first hours after ROSC. Since the liver and kidneys as well as other organs preferentially take up lactic acid during hyperlactatemia, these organs play a key role in rapidly correcting the metabolic acidosis, whether after severe exertion, seizures or OHCA [4–7, 16]. Once lactic acid used a fuel and oxidized to CO_2_ the lungs excrete this CO_2._ Recent studies with labelled lactate show that also under less extreme circumstances, lactate is the main contributor to the tricyclic acid cycle, underscoring lactate’s key role in shuttling energy between organ systems [23]. In a clinical trial in patients with acute heart failure infusion of a large dose of sodium lactate improved cardiac output [24], again indicating that preferential lactate consumption by tissues may confer multiple benefits.

In patients who present with marked hyperlactatemia after OHCA, lactate decreased rapidly in the initial hours indicating the rapid clearance of a massive lactate load through oxidative or gluconeogenetic pathways. Lactate decreased faster than glucose in the early post-OHCA phase underscoring lactate’s preferential metabolism when it is available.

## Limitations

Our study has several limitations. We performed a retrospective study with specific inclusion criteria, such as an initial lactate of ≥ 8 mmol/L in order to select patients with marked hyperlactatemia. We also lacked detailed data on the pre-hospital phase of the cardiac arrest. Also, it was inevitable that the serial measurements of lactate were not performed at the exact same time points. That some patients had more measurements of lactate and glucose (e.g., non-survivors) may have been influenced by various diagnostic and therapeutic actions as probably reflected by the fact that hospital non-survivors had more measurements than survivors.

## Supplementary Information

**Additional file 1: Table S1.** Patient characteristics. **Table S2.** Lactate kinetic parameters in survivors and non-survivors. **Table S3.** Glucose kinetic parameters in survivors and non-survivors. **Figure S1.** Time courses of lactate and glucose in survivors and non-survivors. **Figure S2.** Time courses of pH after OHCA in survivors and non-survivors. **Figure S3.** Time courses of pCO2 after OHCA in survivors and non-survivors. **Figure S4.** Time courses of base excess after OHCA in survivors and non-survivors. **Figure S5.** Individual curves of lactate after OHCA in survivors and non-survivors. **Figure S6.** Individual curves of glucose after OHCA in survivors and non-survivors. **Figure S7.** Individual curves of pH after OHCA in survivors and non-survivors. **Figure S8.** Individual curves of pCO2 after OHCA in survivors and non-survivors. **Figure S9.** Individual curves of base excess after OHCA in survivors and non-survivors.

## Data Availability

Subsets of the datasets used and/or analyzed during the current study are available from the corresponding author on reasonable request.

## Abbreviations

ED: Emergency department
ICU: Intensive care unit
OHCA: Out-of-hospital cardiac arrest
PEA: Pulseless electrical activity
pVT: Pulseless ventricular tachycardia
ROSC: Return to spontaneous circulation
SEM: Standard error of the mean
VF: Ventricular fibrillation
