# Supplementary material for: Early lactate and glucose kinetics following return to spontaneous circulation after out-of-hospital cardiac arrest
Source: BMC Res Notes. 2021 May 13;14:183. doi: 10.1186/s13104-021-05604-w (PMC8120923; doi:10.1186/s13104-021-05604-w)
Supplement: Supplementary file 1 — Additional file 1: Table S1. Patient characteristics. Table S2. Lactate kinetic parameters in survivors and non-survivors. Table S3. Glucose kinetic parameters in survivors and non-survivors. Figure S1. Time courses of lactate and glucose in survivors and non-survivors. Figure S2. Time courses of pH after OHCA in survivors and non-survivors. Figure S3. Time courses of pCO2 after OHCA in survivors and non-survivors. Figure S4. Time courses of base excess after OHCA in survivors and non-survivors. Figure S5. Individual curves of lactate after OHCA in survivors and non-survivors. Figure S6. Individual curves of glucose after OHCA in survivors and non-survivors. Figure S7. Individual curves of pH after OHCA in survivors and non-survivors. Figure S8. Individual curves of pCO2 after OHCA in survivors and non-survivors. Figure S9. Individual curves of base excess after OHCA in survivors and non-survivors. [file 13104_2021_5604_MOESM1_ESM.pdf]

Additional file 1

Accompanying *'Early lactate and glucose kinetics following return to spontaneous circulation after out-of-hospital cardiac arrest'*
by Freire Jorge et al.

Contents

Table S1:	Patient characteristics.
Table S2:	Lactate kinetic parameters in survivors and non-survivors
Table S3:	Glucose kinetic parameters in survivors and non-survivors.
Figure S1:	Time courses of lactate and glucose in survivors and non-survivors.
Figure S2:	Time courses of pH after OHCA in survivors and non-survivors.
Figure S3:	Time courses of pCO_2 after OHCA in survivors and non-survivors.
Figure S4:	Time courses of base excess after OHCA in survivors and non-survivors.
Figure S5:	Individual curves of lactate after OHCA in survivors and non-survivors.
Figure S6:	Individual curves of glucose after OHCA in survivors and non-survivors.
Figure S7:	Individual curves of pH after OHCA in survivors and non-survivors.
Figure S8:	Individual curves of pCO_2 after OHCA in survivors and non-survivors.
Figure S9:	Individual curves of base excess after OHCA in survivors and non-survivors.

Table S1
Patient characteristics.

	All (N=155)	Hospital survivors (N=82)	Hospital non-survivors (N=73)	P-value
Age, mean (SD)	59 (14)	58 (13)	60 (15)	0.45
Male, no. (%)	125 (81%)	72 (88%)	53 (73%)	0.02
Diabetes mellitus, no. (%)	19 (12%)	9 (11%)	10 (14%)	0.63
BLS before ambulance arrival, no. (%)	106 (68%)	58 (72%)	48 (67%)	0.60
Intra-aortic balloon pump, no. (%)	28 (18%)	14 (17%)	14 (19%)	0.84
Initial rhythm, no. (%)				
VF	126 (81%)	75 (92%)	51 (70%)	
pVT	8 (5%)	4 (5%)	4 (6%)	
PEA	8 (5%)	1 (1%)	7 (10%)	< 0.01.
Asystole	11 (7%)	0 (0%)	11 (15%)	
Unknown	2 (1%)	2 (2%)	0 (0%)	

Legend: Characteristics of the study population.

Table S2
Lactate parameters in hospital survivors and non-survivors.

	Hospital survivors (n=82)		Hospital non-survivors (n=73)		Between-group difference		P-value
	Mean	95% CI	Mean	95% CI	Mean	95% CI	
Initial value (mmol/L)	12.4	11.4 to 12.9	12.2	11.2 to 13.1	0.04	-1.2 to 1.2	0.951
Measurements per patient within 8h	5.7	5.6 to 5.9	6.6	6.3 to 6.8	0.9	0.6 to 1.1	<0.001
Time to reach 50% of the initial value (hours)[a]	1.2	1.0 to 1.4	1.8	1.4 to 2.3	0.6	0.09 to 1.1	
Absolute decrease over 8h (mmol/L)	9.8	8.9 to 10.7	8.0	7.1 to 9.0	1.7	0.4 to 3.0	0.010
Relative decrease over 8h (%)	79	74 to 84	67	61 to 72	12	5 to 19	0.002

Legend: Survivors and non-survivors had similar initial lactate levels, but survivors had significantly faster decreases of lactate compared to non-survivors. [a] Basic 95% confidence interval obtained after bootstrapping the mixed-effect model for a thousand times.

Table S2
Glucose parameters in hospital survivors and non-survivors.

	Hospital survivors (n=82)		Hospital non-survivors (n=73)		Between-group difference		P-value
	Mean	95% CI	Mean	95% CI	Mean	95% CI	
**Initial value (mmol/L)**	**17.8**	**16.7 to 18.8**	**19.9**	**18.6 to 21.2**	**2.1**	**0.5 to 3.8**	**0.011**
**Measurements per patient within 8h**	**6.7**	**6.6 to 6.9**	**7.4**	**7.2 to 7.7**	**0.7**	**0.5 to 1.0**	**<0.001**
**Time to reach 50% of the initial value (hours)[a]**	**6.9**	**4.7 to 8.3**	**7.4**	**5.6 to 8.6**	**0.2**	**-2.3 to 3.1**	
**Absolute decrease over 8h (mmol/L)**	**10.6**	**9.2 to 12.0**	**9.9**	**8.5 to 11.2**	**0.7**	**-1.2 to 2.6**	**0.452**
**Relative decrease over 8h (%)**	**51**	**46 to 57**	**53**	**47 to 58**	**1.2**	**-9.5 to 7.2**	**0.784**

Legend: Survivors had a slightly less pronounced hyperglycemia at presentation. The rate of decrease did not differ between survivors and non-survivors. [a]Basic 95% confidence interval obtained after bootstrapping the mixed-effect model for a thousand times.

Figure S1
Kinetics of lactate (a) and glucose (b) and survival

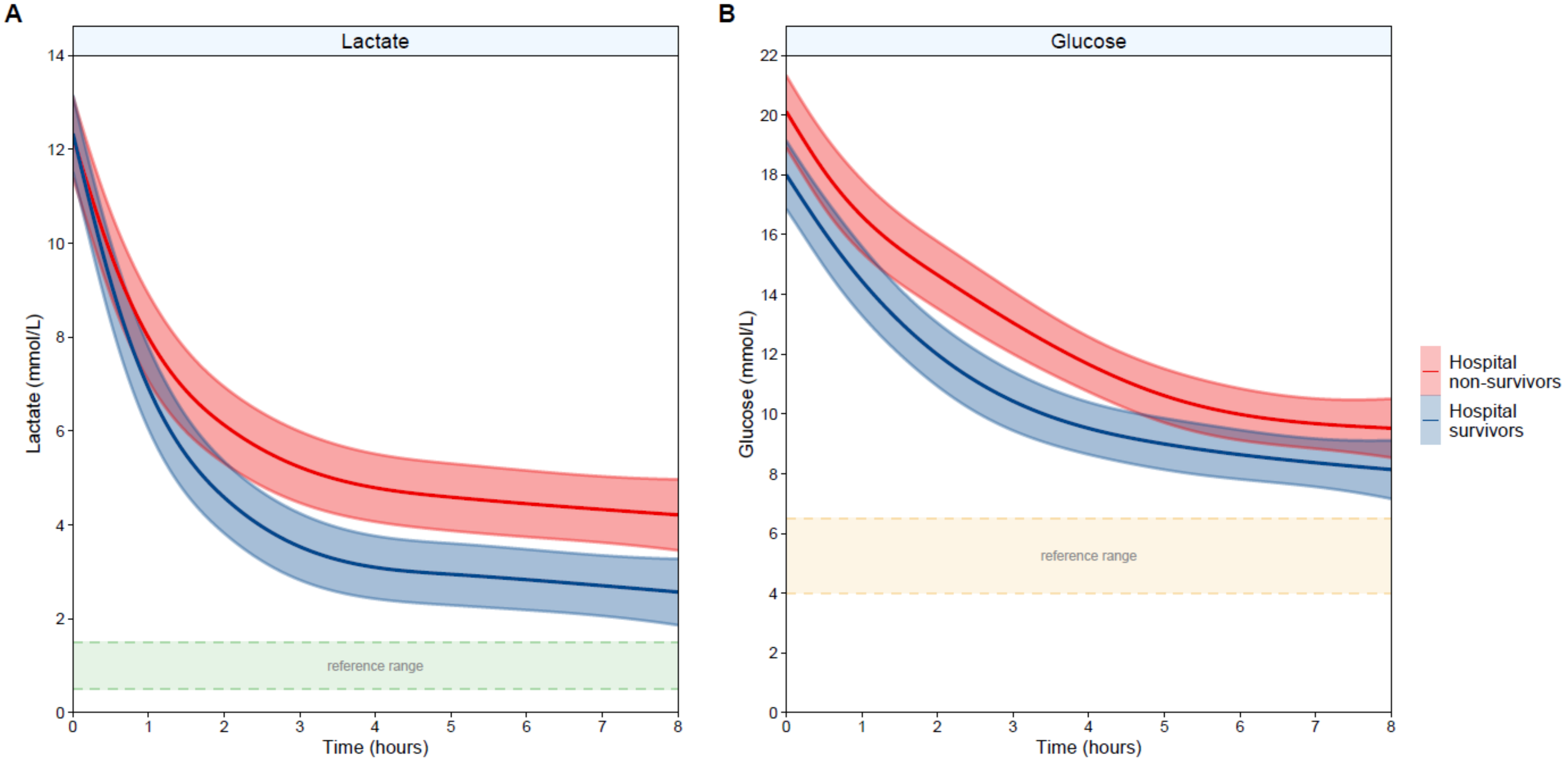

Figure S1: Evolution of lactate (a) and glucose (b) levels during the first 8h after OHCA for hospital survivors and hospital non-survivors. In the case of lactate, most of the decrease occurs in the initial 3h. In the case of glucose, the substantial decrease occurs more gradually during the first 6h. Shaded area indicates 95% confidence interval.

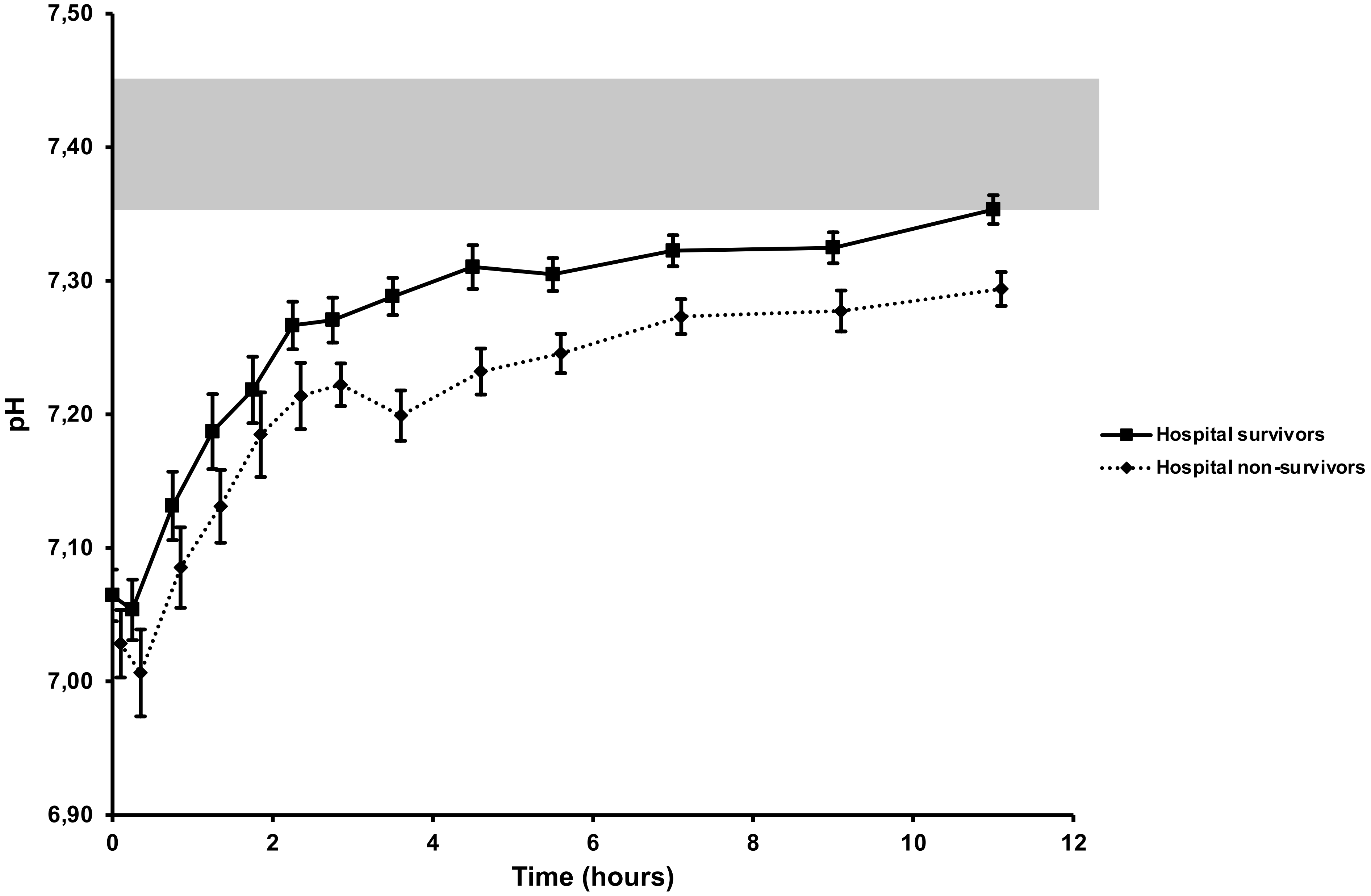

Figure S2: Kinetics of pH after OHCA. Error bars indicate the standard error of the mean. The gray area denotes the reference interval.

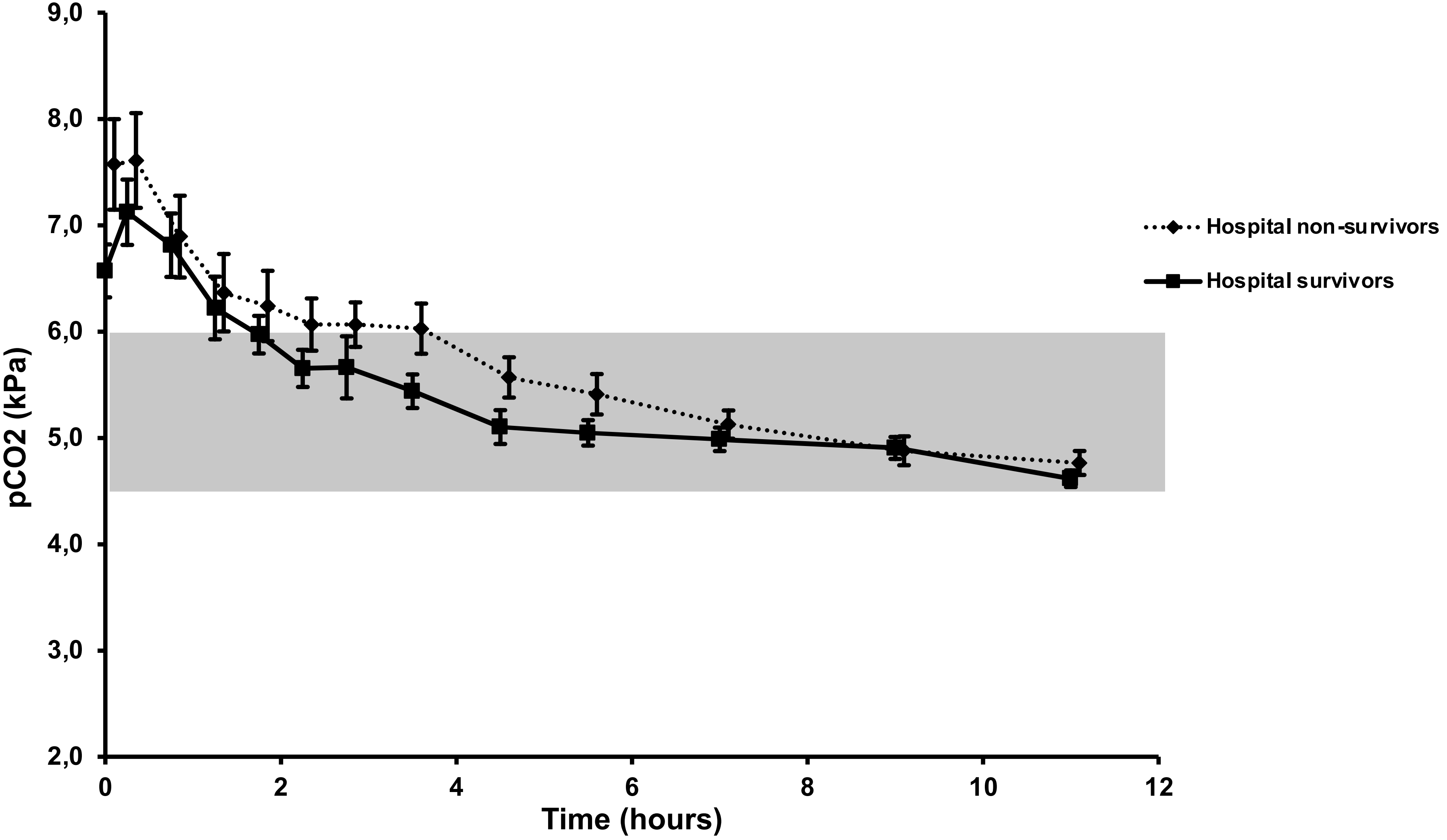

Figure S3: Kinetics of pCO2 after OHCA. Error bars indicate the standard error of the mean. The gray area denotes the reference interval

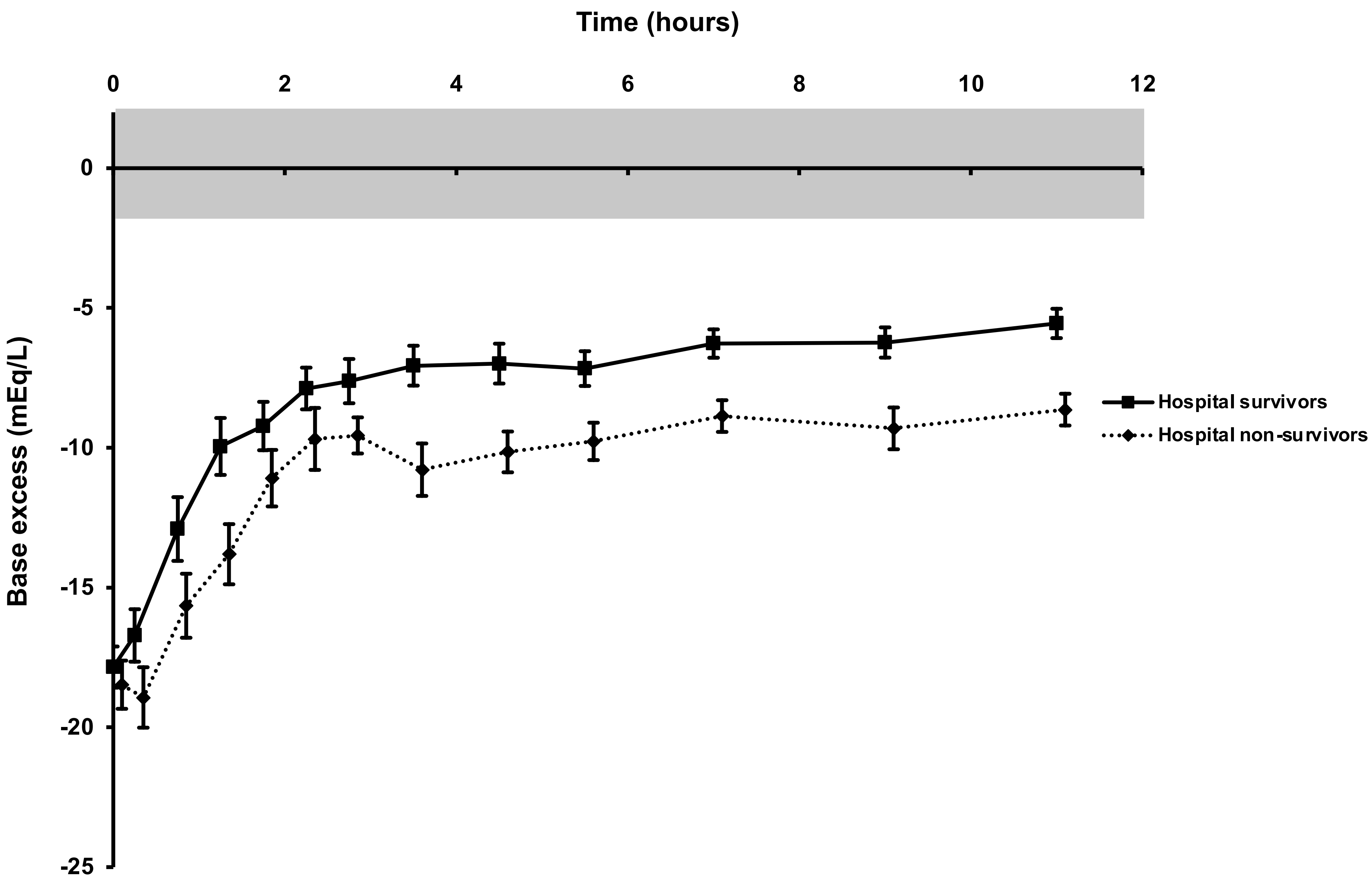

Figure S4: Kinetics of base excess after OHCA. Error bars indicate the standard error of the mean. The gray area denotes the reference interval

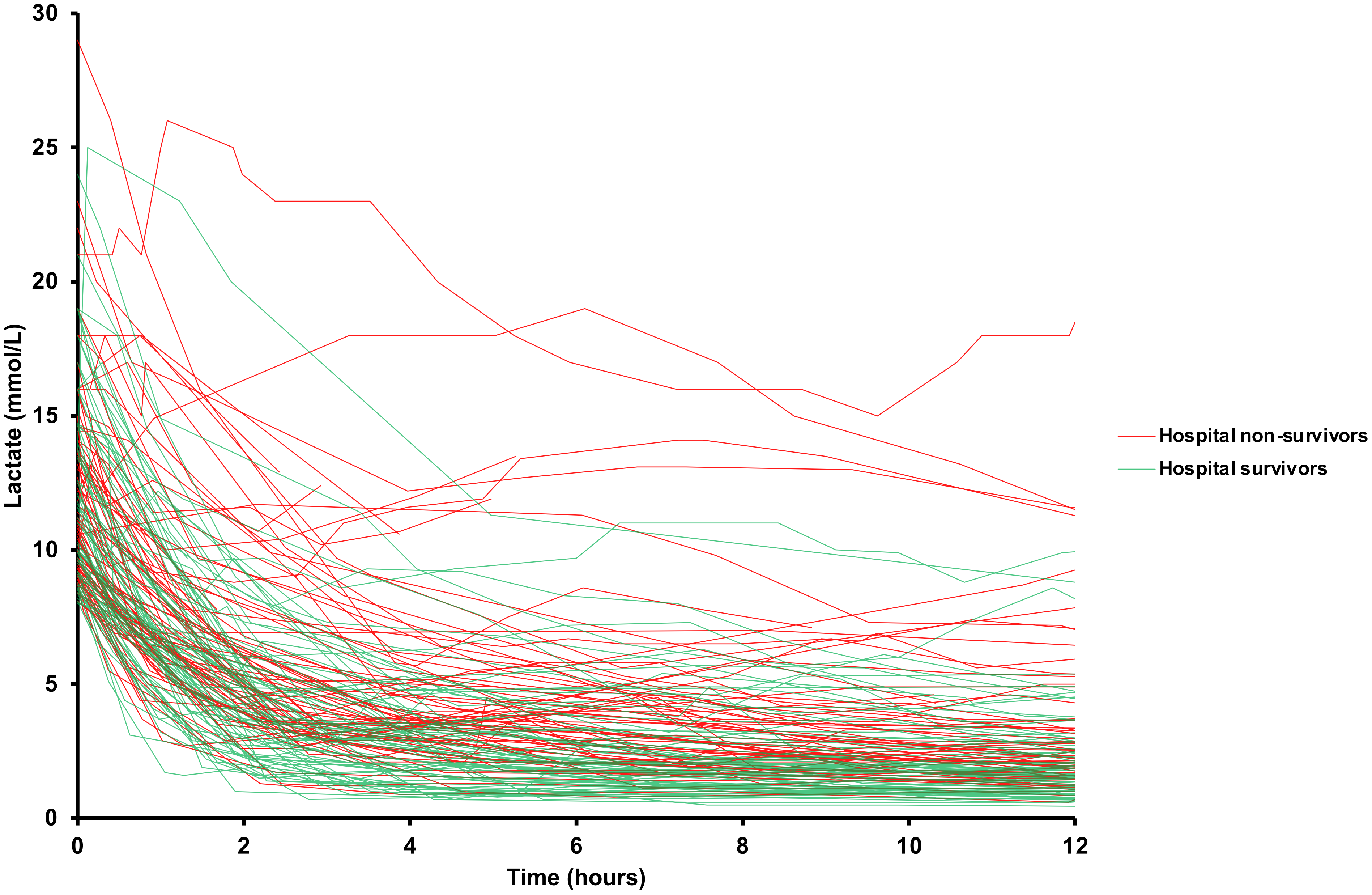

Figure S5: Individual curves of lactate after OHCA. Each line represents a single patient.

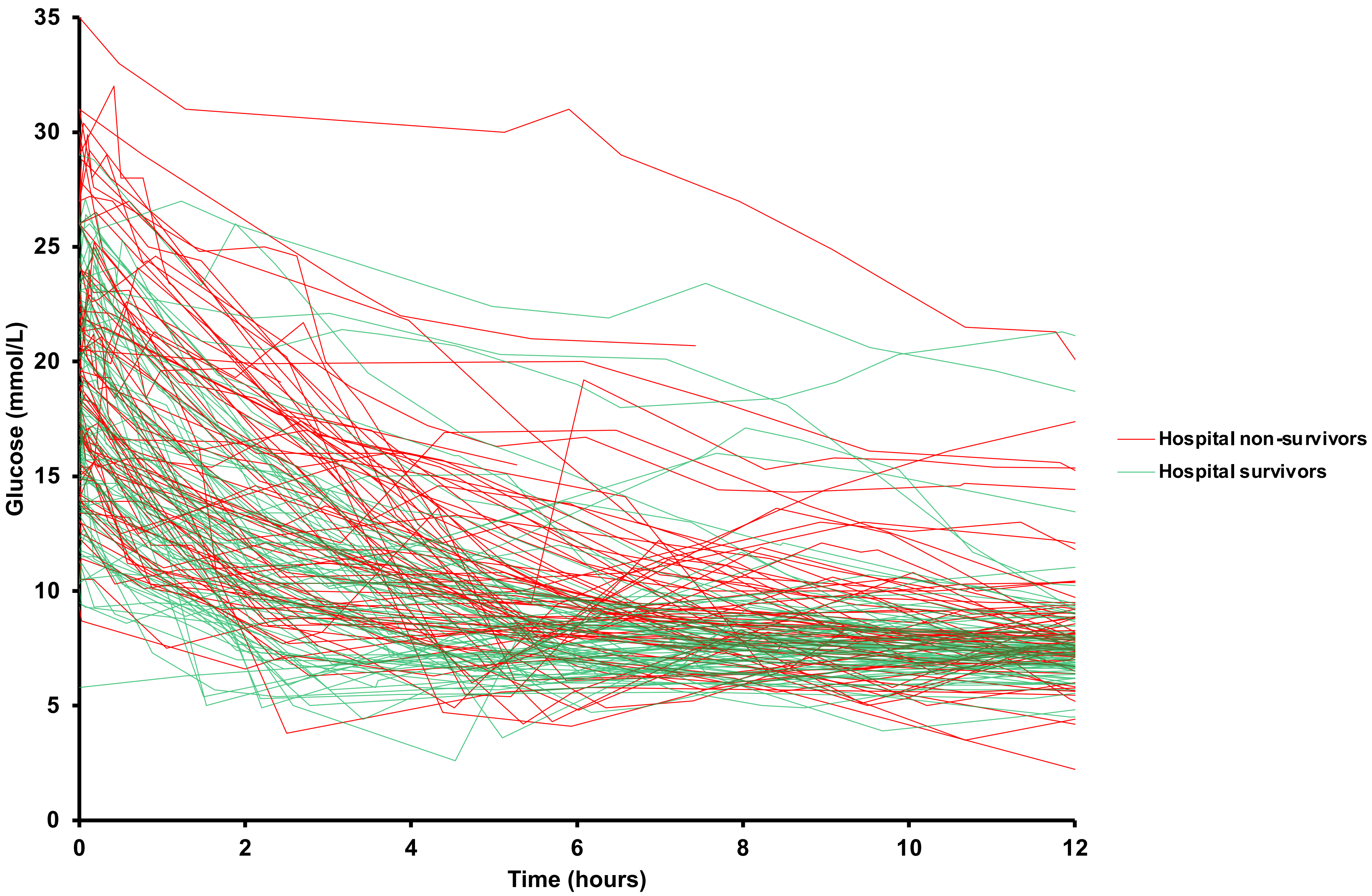

Figure S6: Individual curves of glucose after OHCA. Each line represents a single patient.

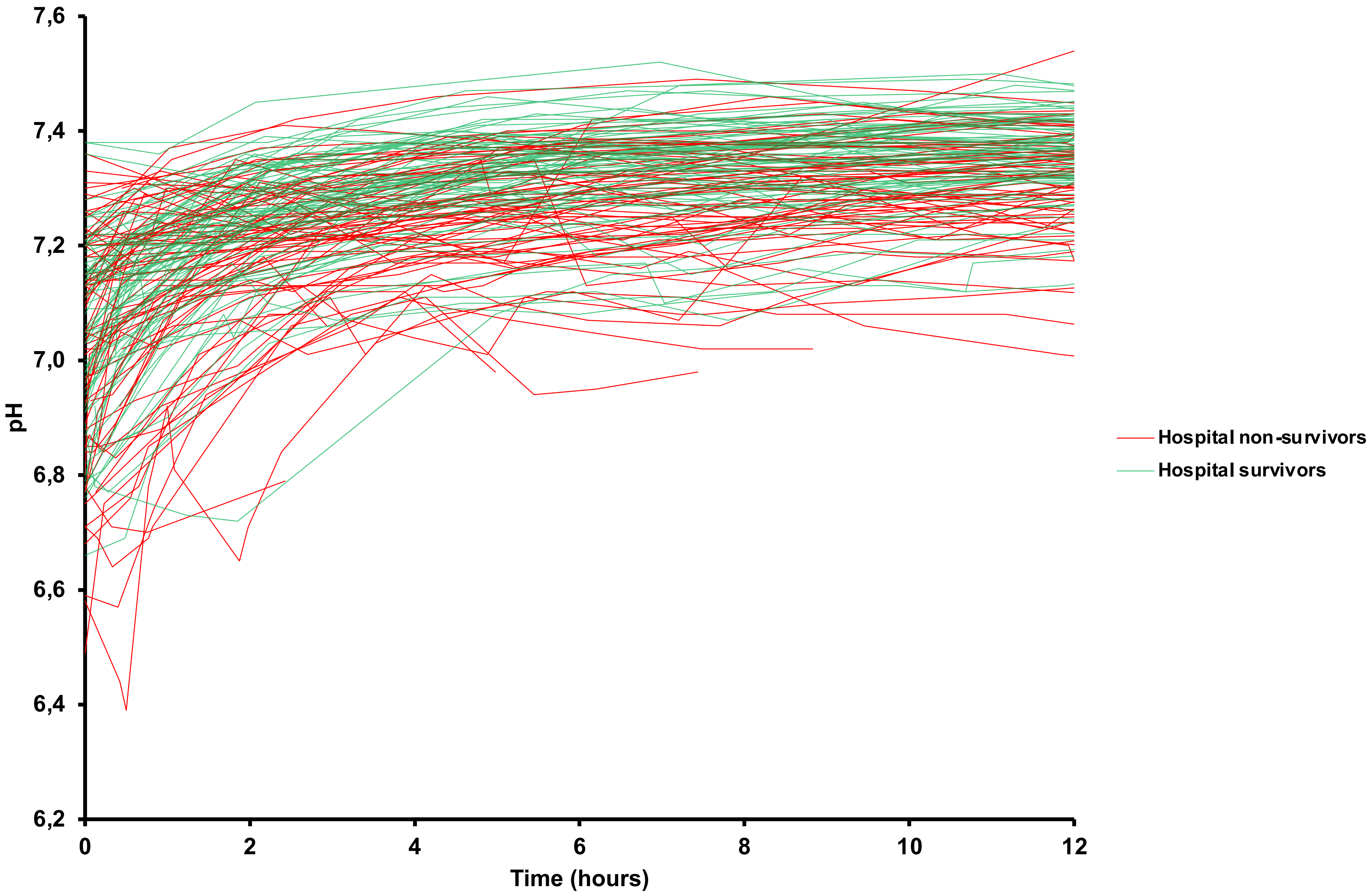

Figure S7: Individual curves of pH after OHCA. Each line represents a single patient.

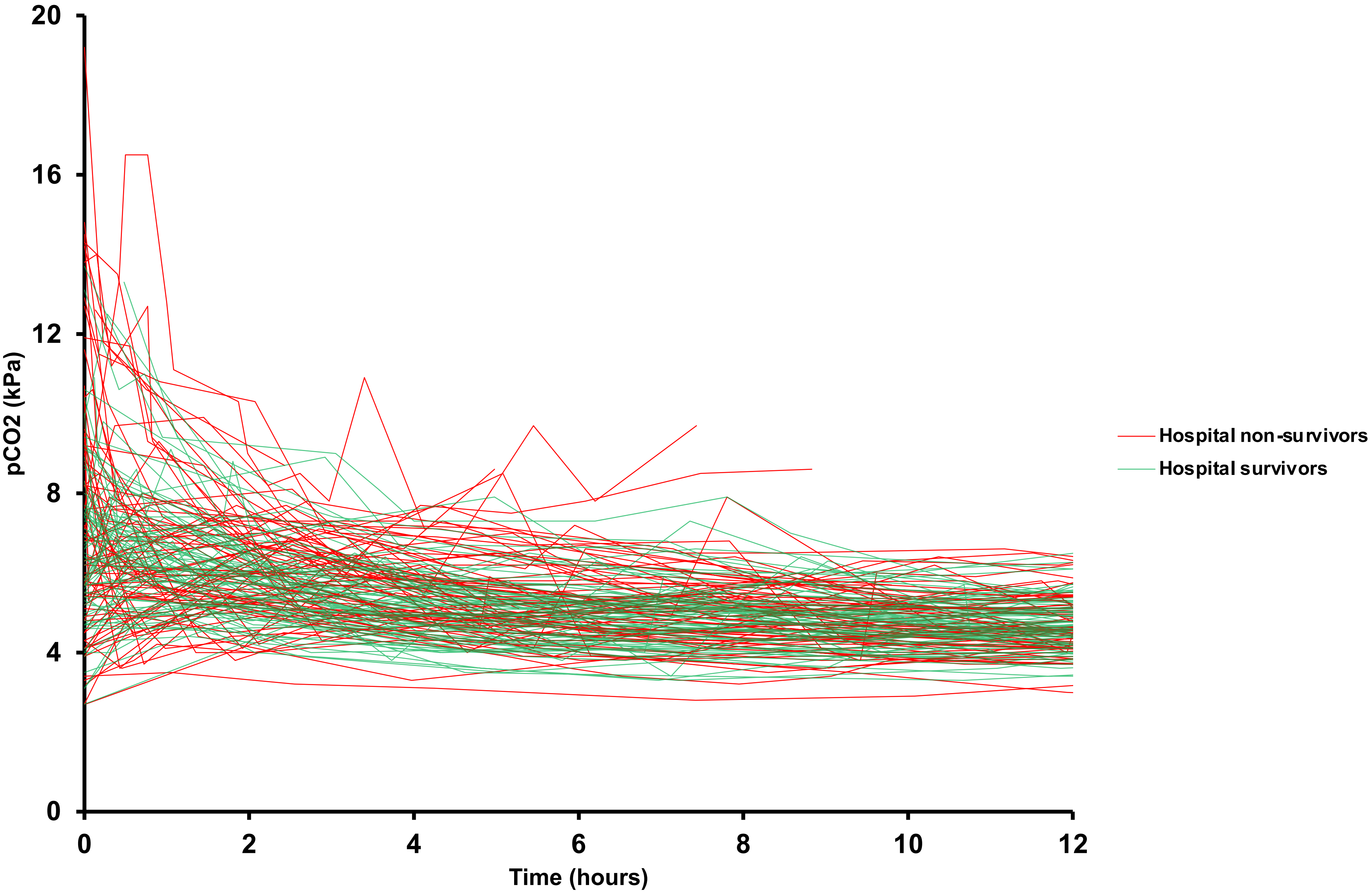

Figure S8: Individual curves of pCO2 after OHCA. Each line represents a single patient.

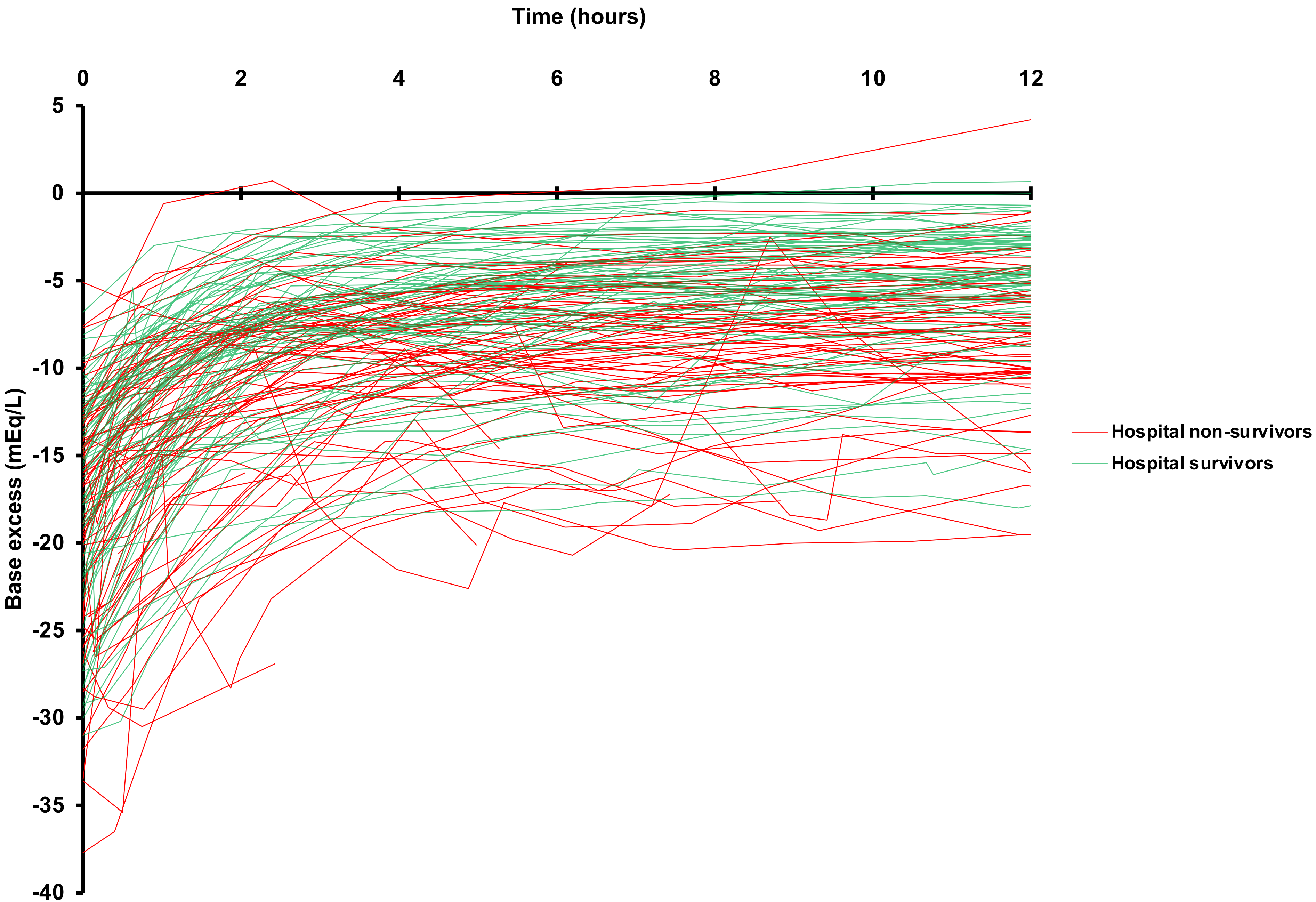

Figure S9: Individual curves of base excess after OHCA. Each line represents a single patient.
